# Establishment of an animal model of ejaculatory duct obstruction

**DOI:** 10.1111/and.14499

**Published:** 2022-06-18

**Authors:** Jianchao Ren, Zhenya Xing, Yuan Ji, Ke Yang, Yu Gao, Wei Wang, Shuaishuai Fan, Jingqi Wang

**Affiliations:** ^1^ Shanxi Medical University Taiyuan China; ^2^ Department of Urology Second Hospital of Shanxi Medical University Taiyuan China

**Keywords:** animal models, ejaculatory duct obstruction, infertility, seminal vesicle, transurethral resection of the ejaculatory duct

## Abstract

This study aimed to establish animal models with different degrees of ejaculatory duct obstruction. Forty‐eight male rats aged 14–15 weeks were randomly divided into three groups (*n* = 16): control, complete ejaculatory duct obstruction (tied around the lower seminal vesicle gland and ductus deferens with a 2–0 silk ligature), and partial ejaculatory duct obstruction (padded with a wire guide). Mortality, complications, seminal vesicle morphology and histopathology were compared in the three groups at 4 and 8 weeks postoperatively. In the complete ejaculatory duct obstruction group, seminal vesicle weight decreased gradually with increased obstruction time compared with those of the control group (*p* < 0.05); moreover, stone‐like material was occasionally observed. In the partial ejaculatory duct obstruction group, there was an increase followed by a decrease in seminal vesicle weight in the postoperative period compared with that of the control group (*p* < 0.05). Histopathological lesions of seminal vesicles were observed in the complete and partial ejaculatory duct obstruction groups (8 weeks postoperatively). We successfully established animal models of complete and partial ejaculatory duct obstruction, which provide an easy‐to‐use tool for studying seminal vesicle changes after ejaculatory duct obstruction.

## 1 INTRODUCTION

Ejaculatory duct obstruction (EDO) is an abnormal lesion of the distal ejaculatory duct of the seminal tract, resulting in sperm discharge obstruction, which accounts for 1%–5% of all causes of male infertility (Achermann & Esteves, 2021). EDO can be categorized as complete or partial depending on the degree of obstruction. EDO not only affects fertility but also causes symptoms, such as weak ejaculation, painful ejaculation, haemorrhage, perineal pain and even psychosocial problems (Parnham & Serefoglu, 2016). Currently, routine semen analysis and transrectal ultrasound are the preferred screening methods for the diagnosis of EDO, given their low invasiveness and simplicity. In addition, several special diagnostic methods are used for EDO, such as endorectal magnetic resonance imaging, seminal vesiculography, technetium Tc 99m sulphur colloid seminal vesicle scintigraphy and ejaculatory duct open pressure measurement (Guo et al., 2013; Modgil, Rai, Ralph, & Muneer, 2016; Orhan et al., 2008). At present, the main treatment for EDO is minimally invasive surgery. Transurethral resection of the ejaculatory duct (TURED) is a classic surgical procedure with a definite curative effect and was first described by Farley and Barnes (Farley & Barnes, 1973). With the development of technology, new minimally invasive surgical procedures are available to treat EDO, including transurethral incision of the ejaculatory duct, transurethral seminal vesiculoscopy and transurethral balloon dilation (Chen et al., 2018; Kayser et al., 2012; Wang, Xie, Zheng, & Jiang, 2021). A systematic review of TURED's efficacy reported that significant improvement in semen volume was observed in 83.0% of EDO patients in 23 studies, with a post‐operative natural pregnancy rate of 25% (Mekhaimar et al., 2020). El‐Assmy, El‐Tholoth, Abouelkheir, & Abou‐El‐Ghar (2012) reported improved semen parameters and natural pregnancy rate in 23 EDO patients after TURED, and achieved better response with partial EDO and cyst‐induced complete EDO. In view of the phenomenon that some EDO patients have no benefit after surgery and different subgroups of EDO patients have different surgical responses, we suspect that EDO causes seminal vesicle lesions. Therefore, it was necessary to establish an animal model that replicates the mechanism of human EDO.

This study aimed to establish an easy‐to‐use animal model of EDO (both complete and partial) to assess mortality, complications, seminal vesicle morphology and histopathology. We hope this model will provide andrologists with a powerful tool to study the pathophysiology of EDO, thereby helping to improve overall male fertility.

## 2 MATERIALS AND METHODS

### 2.1 Animal model preparation

Forty‐eight male Wistar rats (weighing 350–400 g, 14–15 weeks old) from the Animal Experiment Centre of Shanxi Medical University were used in this study. All rodents were housed at a comfortable temperature (23 ± 2°C) and light (12 h light and 12 h dark) with free access to a standard diet and clean water before and after surgery. All surgeries were performed by a single experienced surgeon to ensure uniformity and reproducibility of the animal model. All rats were anaesthetized intraperitoneally using sodium pentobarbital (50 mg/kg, Sheng xing Biotechnology Co Ltd.) before the procedure. The rats were randomly divided into three groups (*n* = 16 per group): the complete EDO group underwent complete bilateral ligation of the lower seminal vesicle gland and ductus deferens, the partial EDO group underwent partial bilateral ligation of the lower seminal vesicle gland and ductus deferens, and the control group only underwent abdominal opening and closing.

### 2.2 Surgical procedures

**FIGURE 1:**
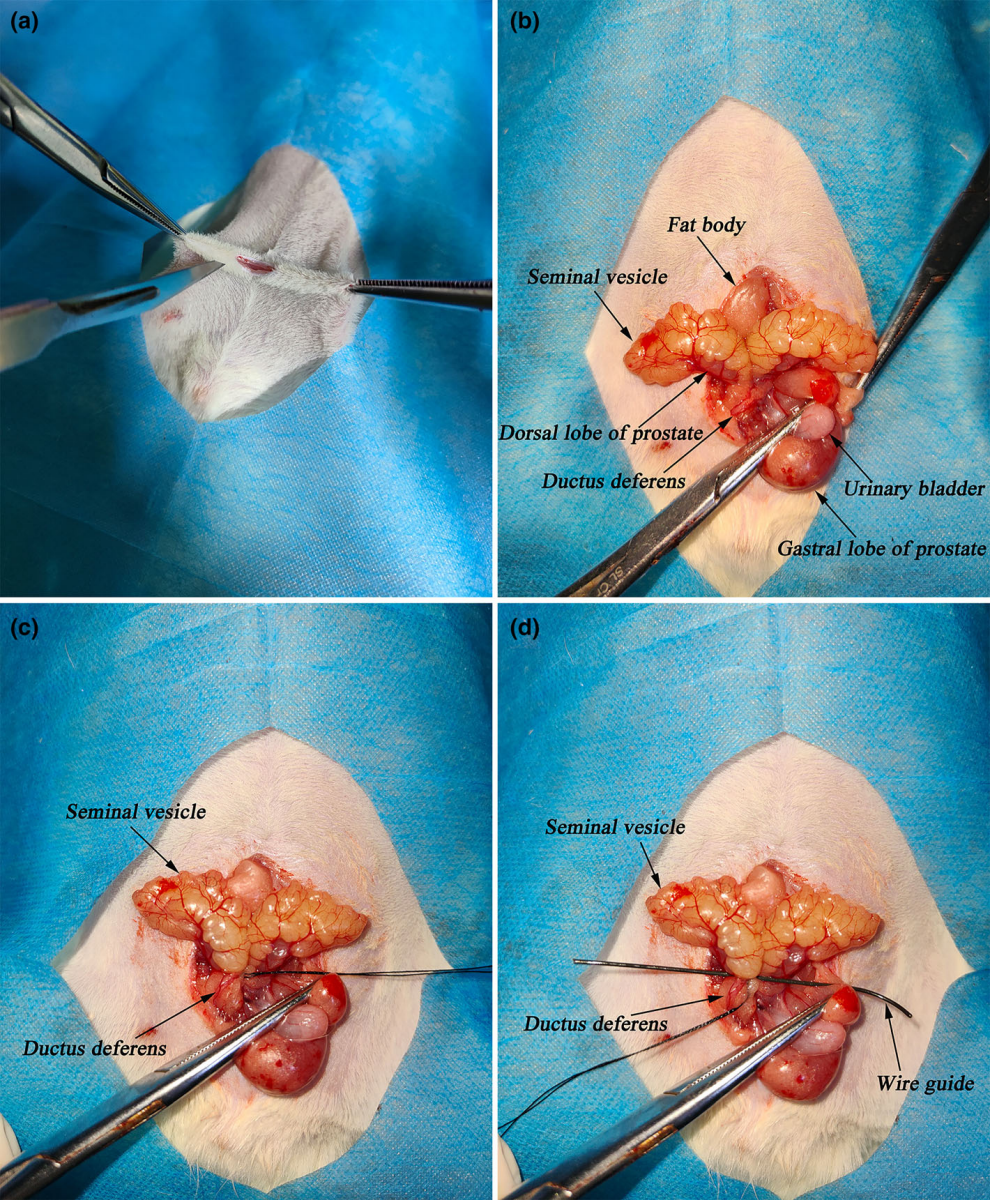
Major surgical procedures. (a) A 4‐cm midline abdominal incision. (b) The tissues surrounding the ejaculatory duct were carefully exposed. (c) The complete EDO group: a 2‐0 silk ligature was tied around the lower seminal vesicle gland and ductus deferens. (d) The partial EDO group: the ligature was padded with a 0.7‐mm wire guide. EDO, ejaculatory duct obstruction.

All animals were fasted for 8 h before surgery. The surgical procedure is illustrated in Figure 1. A brief overview followed, the rats were placed in a supine position on an animal handling platform. The abdomen was shaved, washed and disinfected with povidone‐iodine (Lircon Medical Technology Ltd.). A 4‐cm midline abdominal incision was made, and the fat body, seminal vesicles, urinary bladder and gastral lobe of the prostate were carefully isolated from the surrounding tissue. After carefully dissecting the lower part of the seminal vesicle gland and the gastral lobe of the prostate, the bilateral ductus deferens entered the prostate in a “Y” shape. To create a complete EDO, a 2‐0 silk (Jiasheng Medical Supplies Ltd.) ligature was tied around the lower seminal vesicle gland and ductus deferens. To create a partial EDO, the ligature was padded with a 0.7‐mm wire guide; one end of the ligature was connected to the tension transducer (JZ101, Singapore Airlines Xingye Technology & Trade Ltd.) and the other end was gently and slowly pulled until the tension reached 150 g. Finally, the wire guide was removed, and the abdominal incision was then closed.

Mortality, complications, seminal vesicle morphology and histopathology were evaluated at 4 weeks postoperatively in half of the rats and the remainder at 8 weeks.

### 2.3 Histopathological study

Half of the rats in each group were humanely sacrificed 4 weeks after surgery, and the seminal vesicles were rapidly removed and weighed. The remaining rats in each group received the same treatment 8 weeks after surgery. The ratio of bilateral seminal vesicle weight (mg) to body weight (g) was calculated for all rats. Equal‐sized specimens from each group of seminal vesicles were cut and instantly fixed with 4% paraformaldehyde. The tissues were embedded in paraffin and sectioned (5 μm). Haematoxylin and eosin staining was performed to examine tissue histopathological changes.

### 2.4 Statistical analysis

All experiments were performed in triplicate. The analysis was performed using SPSS Statistics 26.0 software (IBM). The data are expressed as mean ± SD, and the differences between groups were compared using analysis of variance; for all statistical tests, *p* < 0.05 was considered to be significant.

## 3 RESULTS

### 3.1 Establishment of the model

One animal in the complete EDO group died of postoperative wound infection, whereas two animals died in the partial EDO group due to postoperative wound infection and intestinal obstruction. No complications occurred in the other animals. In the surviving animals, obvious modelling effects were observed in the complete EDO group; two rats in the partial EDO group were excluded due to the loss of ligature knot. The operation time, survival rate and modelling success rate of each group are found in Table 1.

**TABLE 1:** Results of operation time, survival rate and modelling success rate in each group (*n* = 16)

Groups	Operative time (min)	Survival rate (%)	Modelling success rate (%)
Complete EDO	14.9 ± 1.37	93.75	100.00
Partial EDO	18.2 ± 1.03	87.50	85.71
Control	9.55 ± 1.05	100.00	100.00

*Note*: Values are presented as mean ± standard deviation (Mean ± SD).

Abbreviation: EDO, ejaculatory duct obstruction.

### 3.2 Gross morphology

**FIGURE 2:**
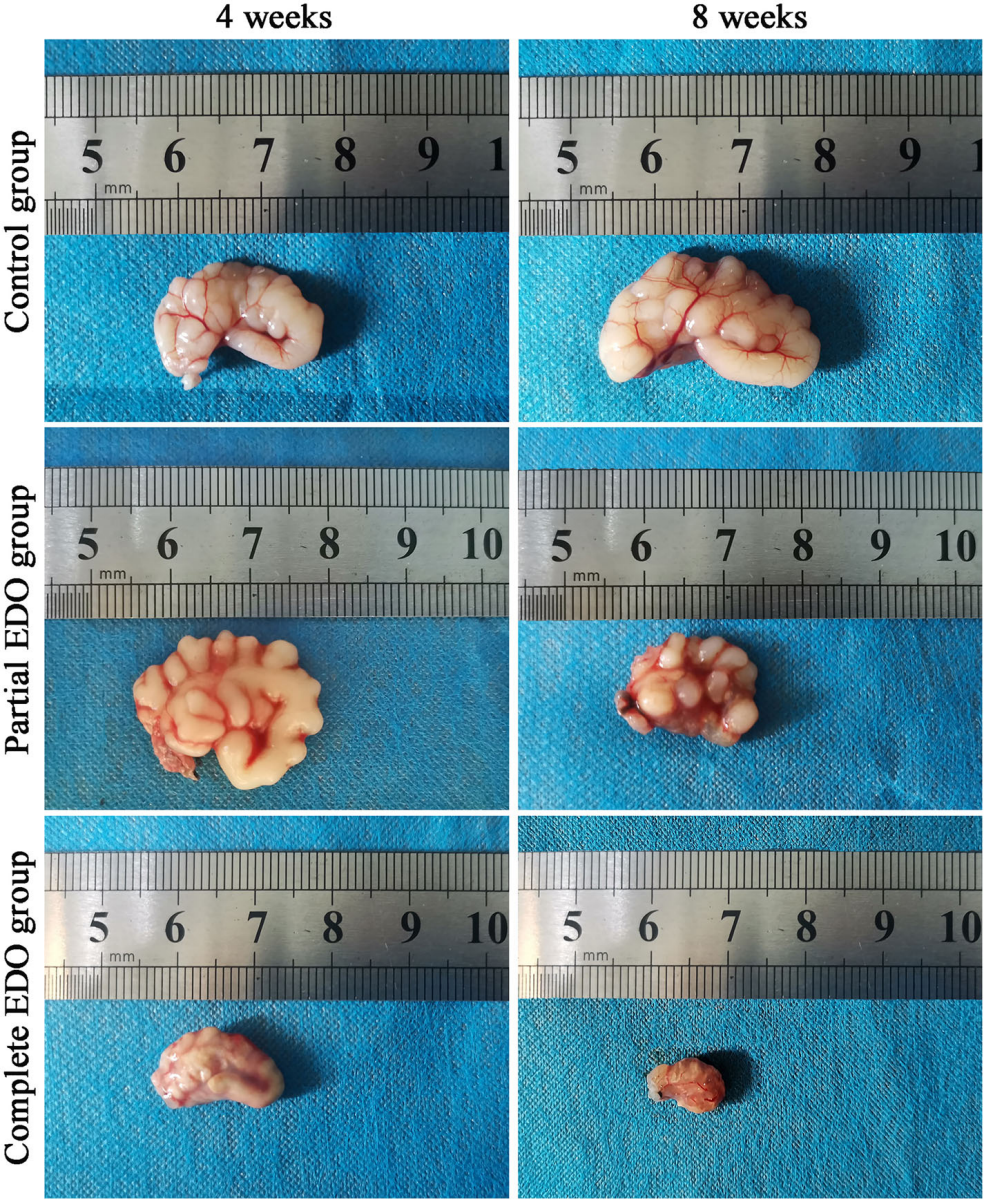
Gross seminal vesicle morphology of the complete and partial EDO groups, and that of the control group. EDO, ejaculatory duct obstruction.

The seminal vesicles were removed and photographed (Figure 2). The seminal vesicles in the complete EDO group were atrophied and hardened, and their contents coagulated, which worsened with the duration of obstruction. Stone‐like material was visible in the seminal vesicle contents of some animals in the complete EDO group 8 weeks postoperatively, and infrared spectroscopy of stone composition showed apatite carbonate (9/10) and calcium oxalate monohydrate (1/10). In the partial EDO group, the seminal vesicles were larger and fuller 4 weeks postoperatively than the seminal vesicles in the control group, and some of the glandular cavities were atrophied and hardened 8 weeks postoperatively.

### 3.3 Body and organ weights

**FIGURE 3:**
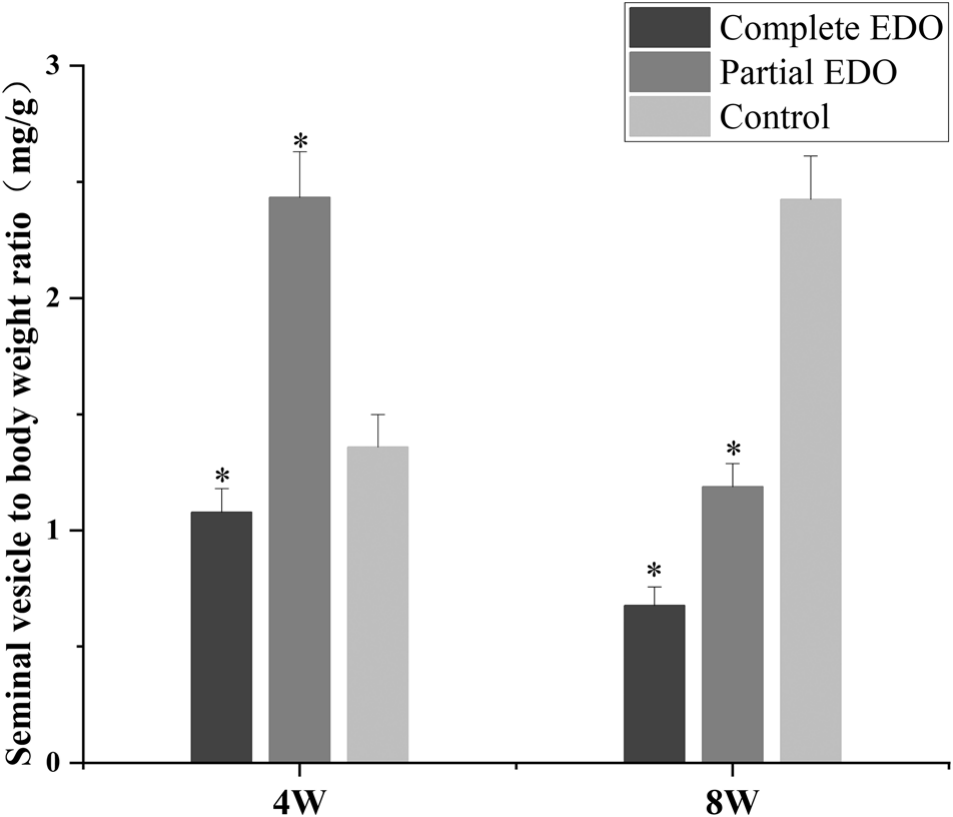
Seminal vesicle weights (mg) normalized to body weight (g) at 4 and 8 weeks postoperatively. The asterisk indicates *p* < 0.05 compared with the control group.

There was no significant difference in body weight between the groups at 4 and 8 weeks after surgery. The ratio of the seminal vesicle weight (mg) to body weight (g) can be used to verify changes in the seminal vesicle weight and whether the histopathological change was caused by the surgery (Figure 3). At 4 and 8 weeks after surgery, the weight of the seminal vesicles was significantly lower in the complete EDO group (ratio: 1.08 ± 0.10 at 4 weeks and 0.68 ± 0.08 at 8 weeks after surgery). In the partial EDO group, the weight of the seminal vesicles increased significantly 4 weeks after surgery (ratio: 2.43 ± 0.20) and decreased significantly 8 weeks after surgery (ratio: 1.19 ± 0.10).

### 3.4 Histopathological examination

**FIGURE 4:**
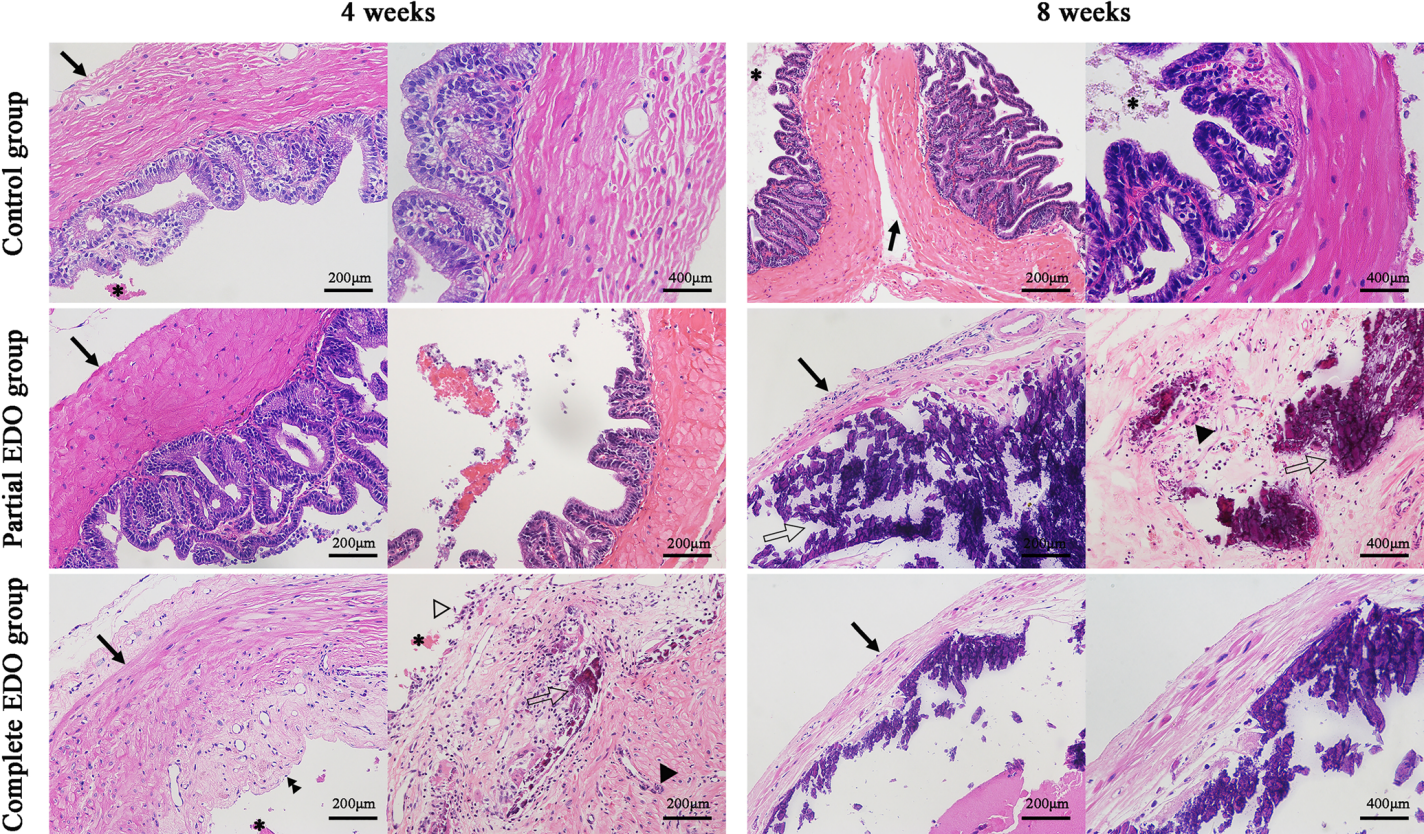
Histopathological examination of seminal vesicles in the rats at 4 and 8 weeks postoperatively. The control group (4 and 8 weeks): a large glandular lumen with occasional eosinophilic secretions (asterisks) is noted; the mucosal folds have an alveolus‐like structure, with a thicker smooth muscle layer (arrows). The partial EDO group at 4 weeks: the smooth muscle thickness is increased (arrow). At 8 weeks: blue‐stained crystalloids (open arrows) and multinucleated giant cells (arrowhead) are seen in the glandular lumen. The smooth muscle layer (arrow) is significantly thinner, with no epithelial cells. The complete EDO group at 4 weeks: the reduced glandular lumen contains more blue‐stained crystalloids (open arrow), multinucleated giant cells (arrowhead), eosinophilic secretions (asterisks), and some degenerated exfoliated epithelial cells (open arrowhead). The smooth muscle layer (arrow) is thinner, and some degenerated epithelial cells (double arrowhead) are observed. At 8 weeks: the smooth muscle layer (arrow) is significantly thinner, and no epithelial cells are observed. EDO, ejaculatory duct obstruction.

Histopathological changes in each group are shown in Figure 4. Four weeks after surgery, the smooth muscle layer appeared to be thickened in partial EDO, but no other histological changes were found. The complete EDO group showed damage, including blue‐stained crystalloids, reduced epithelial cells, a thin smooth muscle layer and multinucleated giant cells. Eosinophilic secretions and degenerated exfoliated epithelial cells were observed in the control group. Eight weeks after surgery, the partial EDO group also showed damage changes, such as thinning of the muscle layer, loss of epithelial cells, blue‐stained crystalloid and multinucleated giant cells. In the complete EDO group, the extent of the damage increased over time, with the smooth muscle layer becoming thinner and the epithelial cells barely visible.

## 4 DISCUSSION

With the rapid development of diagnostic technology and gradual improvement in men's self‐health awareness, andrologists are increasingly encountering EDO patients. Currently, transurethral resection of the ejaculatory duct is the classic surgical method for the treatment of complete and partial EDO. In our long‐term clinical study, we found that the semen of some patients did not improve even when the ejaculatory duct obstruction was removed, a finding that is consistent with the results observed by other researchers (Raheem, De Luca, Muneer, & Ralph, 2016; Sangster et al., 2017; Tu et al., 2013). We hypothesized that EDO leads to increased intracapsular pressure in the seminal vesicles, thus impairing the seminal vesicle.

Few basic studies on seminal vesicle injury caused by EDO have been conducted at home and abroad. We implemented previous modelling methods, whose introduction of tension transducers ensures consistent ligature diameters and guarantees the stability of the partial EDO models (Fei, Wen‐jun, & Xiao‐feng, 2010). In this experiment, the round passivated 10‐mL syringe needle was replaced with a 0.7‐mm wire guide to avoid surgical side injuries. Care should be taken when completely ligating the ejaculatory duct because it may be disconnected. In this experiment, no water was given for 8 h before the procedure to prevent excessive stretching of the bladder and thus made it easier to expose the lower end of the seminal vesicles and ductus deferens, which would otherwise be prone to bladder bleeding or even rupture. It is worth noting that the anatomy of rats differs from that of humans. In humans, the seminal vesicle excretory duct merges with the ampulla of the ductus deferens to form the ejaculatory duct, which passes through the prostate and finally opens into the prostatic urethra at the verumontanum. In rats, the relative length of the ductus deferens is small, and the seminal vesicle excretory duct joins the ductus deferens in the urethra to form the ejaculatory duct. We chose to ligate the lower seminal vesicle gland and ductus deferens rather than directly ligate the ejaculatory duct, which not only had no impact on the study of EDO on seminal vesicle changes but also ensured the establishment of animal models safely and quickly.

Our results showed that EDO leads to progressive damage to the seminal vesicles, which was consistent with our hypothesis. This damage included atrophy of the seminal vesicles, progressive thinning of the muscle layer and a gradual decrease in epithelial cells. Blue‐stained crystalloid and multinucleated giant cells in the glandular lumen appeared because of EDO. Eosinophilic secretions and degenerated exfoliated epithelial cells were not particularly significant in seminal vesicle injury. In the partial EDO group, however, the seminal vesicles compensated for the increased intravesicular pressure 4 weeks after surgery. With the extension of the partial obstruction time, the compensatory mechanism gradually lost its function.

This is the first animal model with different degrees of EDO, and it can lay a theoretical foundation for the pathophysiological changes in seminal vesicles after EDO. However, changes in intracapsular seminal pressure and semen parameters after the release of obstruction were not assessed in this experiment, and thus need to be investigated in future studies. In addition, this study did not explore the mechanisms associated with EDO that led to seminal vesicle alterations. Therefore, follow‐up experiments are needed to investigate the interrelated mechanisms and generate experimental evidence.

## 5 CONCLUSION

We have successfully established an animal model of complete and partial EDO. The model is easy to construct, the effect is precise, and obstruction is easy to cause and release. Moreover, it provides an excellent research tool and method to further study and clarify the pathophysiological changes in seminal vesicles after EDO.

## AUTHOR CONTRIBUTIONS

J Wang contributed to conception and design and administrative support. J Ren, Z Xing, K Yang, Y Gao and W Wang provided study materials. J Ren, Z Xing and Y Ji collected and assembled data. J Ren, S Fan and Y Ji analysed and interpreted data. All authors wrote and did final approval of the manuscript.

## FUNDING INFORMATION

Natural Science Foundation of Shanxi Province, grant number: 201901D111346; Health Science Popularization Project of Shanxi Association for Science and Technology, grant number: JKKP202137.

## CONFLICT OF INTEREST

The authors have no conflicts of interest to declare.

## ETHICS STATEMENT

Experiments were performed by the Animal Research Ethics Committee of the First Hospital of the Shanxi Medical University. Animals were treated in accordance with the National Institutes of Health guide for the care and use of laboratory animals.

## Data Availability

The data that support the findings of this study are available from the corresponding author upon reasonable request.

## References

[and14499-bib-0001] Achermann, A. P. P. , & Esteves, S. C. (2021). Diagnosis and management of infertility due to ejaculatory duct obstruction: Summary evidence. International Brazilian Journal of Urology, 47(4), 868–881. 10.1590/s1677-5538.Ibju.2020.0536 33566474 PMC8321472

[and14499-bib-0002] Chen, R. , Wang, L. , Sheng, X. , Piao, S. G. , Nian, X. W. , Cheng, X. , Zhou, T. , Li, H. Z. , Liu, Y. W. , Chen, G. H. , Zhang, C. L. , Kong, D.‐P. , Xiao, G.‐A. , Lu, X. , Jia, Z.‐Y. , Liu, Z.‐Y. , & Sun, Y. H. (2018). Transurethral seminal vesiculoscopy for recurrent hemospermia: Experience from 419 cases. Asian Journal of Andrology, 20(5), 438–441. 10.4103/aja.aja_76_17 29735816 PMC6116688

[and14499-bib-0003] El‐Assmy, A. , El‐Tholoth, H. , Abouelkheir, R. T. , & Abou‐El‐Ghar, M. E. (2012). Transurethral resection of ejaculatory duct in infertile men: Outcome and predictors of success. International Urology & Nephrology, 44(6), 1623–1630. 10.1007/s11255-012-0253-6 22833254

[and14499-bib-0004] Farley, S. , & Barnes, R. (1973). Stenosis of ejaculatory ducts treated by endoscopic resection. Journal of Urology, 109(4), 664–666. 10.1016/s0022-5347(17)60510-x 4695109

[and14499-bib-0005] Fei, X. , Wen‐jun, B. , & Xiao‐feng, W. (2010). Partial ejaculatory duct obstruction induces hemospermia syndrome in the rat model. Zhonghua Nan Ke Xue, 16(03), 240–243. 10.13263/j.cnki.nja.2010.03.011 20369553

[and14499-bib-0006] Guo, Y. , Liu, G. H. , Yang, D. , Sun, X. Z. , Wang, H. J. , Deng, C. H. , Zhang, Y. , & Feng, S. T. (2013). Role of MRI in assessment of ejaculatory duct obstruction. Journal of X‐Ray Science and Technology, 21(1), 141–146. 10.3233/xst-130361 23507860

[and14499-bib-0007] Kayser, O. , Osmonov, D. , Harde, J. , Girolami, G. , Wedel, T. , & Schafer, P. (2012). Less invasive causal treatment of ejaculatory duct obstruction by balloon dilation: A case report, literature review and suggestion of a CT‐ or MRI‐guided intervention. German Medical Science: GMS e‐journal, 10(4), Doc06. 10.3205/000157 22557939 PMC3334934

[and14499-bib-0008] Mekhaimar, A. , Goble, M. , Brunckhorst, O. , Alnajjar, H. M. , Ralph, D. , Muneer, A. , & Ahmed, K. (2020). A systematic review of transurethral resection of ejaculatory ducts for the management of ejaculatory duct obstruction. Turkish Journal of Urology, 46(5), 335–347. 10.5152/tud.2020.20228 32915715 PMC7483456

[and14499-bib-0009] Modgil, V. , Rai, S. , Ralph, D. J. , & Muneer, A. (2016). An update on the diagnosis and management of ejaculatory duct obstruction. Nature Reviews Urology, 13(1), 13–20. 10.1038/nrurol.2015.276 26620608

[and14499-bib-0010] Orhan, I. , Duksal, I. , Onur, R. , Balci, T. A. , Poyraz, K. , Firdolas, F. , & Kadioğlu, A. (2008). Technetium Tc 99m Sulphur colloid seminal vesicle scintigraphy: A novel approach for the diagnosis of the ejaculatory duct obstruction. Urology, 71(4), 672–676. 10.1016/j.urology.2007.11.103 18313106

[and14499-bib-0011] Parnham, A. , & Serefoglu, E. C. (2016). Retrograde ejaculation, painful ejaculation and hematospermia. Translational Andrology and Urology, 5(4), 592–601. 10.21037/tau.2016.06.05 27652230 PMC5002007

[and14499-bib-0012] Raheem, A. A. , De Luca, F. , Muneer, A. , & Ralph, D. (2016). Presentation and treatment outcome of ejaculatory duct obstruction (EDO). Journal of Sexual Medicine, 13(6), S265. 10.1016/j.jsxm.2016.04.060

[and14499-bib-0013] Sangster, P. , Kalejaiye, A. , Chiriaco, G. , Raheem, A. , Muneer, A. , & Ralph, D. (2017). Presentation and treatment outcomes of ejaculatory duct obstruction. Journal of Urology, 197(4), e1207. 10.1016/j.juro.2017.02.2811

[and14499-bib-0014] Tu, X. A. , Zhuang, J. T. , Zhao, L. , Zhao, L. Y. , Zhao, J. Q. , Lu, K. L. , Sun, X. Z. , Qiu, S. P. , Zhang, Y. , & Deng, C. H. (2013). Transurethral bipolar plasma kinetic resection of ejaculatory duct for treatment of ejaculatory duct obstruction. Journal of X‐Ray Science and Technology, 21(2), 293–302. 10.3233/xst-130377 23694916

[and14499-bib-0015] Wang, K.‐N. , Xie, W.‐L. , Zheng, L. , & Jiang, T. (2021). Transurethral vesiculoscope‐assisted laser incision of the prostatic utricle to treat partial ejaculatory duct obstruction. Asian Journal of Andrology, 23(1), 120–121. 10.4103/aja.aja_144_19 32129190 PMC7831826

